# Impact of Age and Hearing Impairment on Work Performance during Long Working Hours

**DOI:** 10.3390/ijerph15010098

**Published:** 2018-01-09

**Authors:** Verena Wagner-Hartl, Nina R. Grossi, K. Wolfgang Kallus

**Affiliations:** 1 Department of Psychology, University of Graz, 8010 Graz, Austria; nina.grossi@jku.at (N.R.G.); wolfgang.kallus@uni-graz.at (K.W.K.); 2 Faculty Industrial Technologies, Campus Tuttlingen, Furtwangen University, 78532 Tuttlingen, Germany; 3 Department of Work Organizational and Media Psychology, Johannes Kepler University Linz, 4040 Linz, Austria

**Keywords:** older employees, reduced hearing capacity, overtime hours, performance

## Abstract

Based on demographic prognoses, it must be assumed that a greater number of older workers will be found in the future labor market. How to deal with their possible age-related impairments of sensory functions, like hearing impairment and work performance during extended working time, has not been addressed explicitly until now. The study addresses this interplay. The study was performed on two consecutive days after normal working hours. The 55 participants had to “work” in the study at least three additional hours to simulate a situation of long working hours. The tested measures for (job) performance were: general attention, long-term selective attention, concentration, and reaction time. All of the investigated variables were taken at both days of the study (2 × 2 × 2 repeated measurement design). The results show effects for age, the interaction of hearing impairment and time of measurement, and effects of the measurement time. Older participants reacted slower than younger participants did. Furthermore, younger participants reacted more frequently in a correct way. Hearing impairment seems to have a negative impact especially on measures of false reactions, and therefore especially on measurement time 1. The results can be interpreted in a way that hearing-impaired participants are able to compensate their deficits over time.

## 1. Introduction

Demographic development raises expectations of an aging in the working population in the next years [1,2]. This prognosis emphasizes the importance of keeping employees healthy and strengthening their ability to work until they reach retirement-age. Furthermore, this aging of the working population implicates dealing with age-related impairments of sensory functions, such as hearing impairment. Moreover, the National Academy on an Aging Society [3] reported a retirement rate of 18% for hearing-impaired, and a retirement rate of 12% for normal hearing American employees aged between 51 and 61 years. Additionally, data of the U.S. working population have shown that 75% of the normal hearing when compared to 67% of the hearing-impaired working-age population was employed. Furthermore, there is evidence for an imbalance of employment between hearing impaired and normally hearing individuals: 30% of hearing impaired adults in Sweden were unemployed as compared to only 12% of normally hearing adults [4].

Following the World Health Organization (WHO) [1], aging workers are defined as employees that are 45 years and older. As workers age and acquire age-related sensory, cognitive, and physical changes, they are faced with increasing challenges in continuing to be productive in the workplace. Hearing impairment is, besides vision-related impairments, one of the prominent symptoms of increasing age. Until now, how to deal with their possible age-related impairments of sensory functions like hearing impairment and work performance during extended working time has not been addressed explicitly. The current paper addresses this interplay based on data from a laboratory study.

### 1.1. Hearing Impairment

Regarding impairments of sensory functions, results of a Finnish survey study [5], as well as of the “Epidemiology of Hearing Loss Study” in Beaver Dam Wisconsin that used standard audiometric testing [6] provide evidence for a high prevalence of hearing impairment for older working-aged persons. Cruickshanks et al. [6] showed that the prevalence of hearing loss increased with age. Further, the WHO [7] estimates that 1.1 billion young people worldwide could also be at risk of hearing loss. This is mainly due to unsafe listening practices and unsafe or damaging levels of sounds (e.g., listening to their audio devices, visiting clubs, bars, or discotheques). Taking account of this fact, research regarding reduced hearing capacity of persons of working age, becomes more and more important even for younger persons.

It has previously been observed that reduced hearing capacity has a significant impact on the quality of life of the concerned persons [3,8,9,10,11,12,13]. Furthermore, uncorrected reduced hearing also influences all other persons who want to communicate with the impaired persons [14,15,16]. In addition, hearing impaired employees reported a higher need of recovery after work [17] and significant more sickness absence or rather sick-leave [18] than normal hearing employees. Interestingly, Kramer et al. [18] showed that hearing-impaired also reported significantly more sick-leave due to mental distress compared to normal hearing employees. Thus, it is obvious that hearing impairment also has a significant impact at work. Moreover, data from several studies suggest negative effects of hearing impairment on (job) performance. These effects are caused by an impairment of speech comprehension, speech perception in noise, memory performance and selective attention esp. auditory object selection, and formation and switching of attention e.g., in complex settings, multi-person conversation, etc. [15,19,20,21,22,23,24,25,26,27,28,29,30,31,32,33,34]. However, the results are complex. Some studies indicated that reduced hearing capacity is not associated with lower cognitive performance (e.g., measured with tests on memory performance, attention, and spatial working memory), especially when nonverbal/non-auditory material is used in cognitive tests [35]. Zekveld, A.A. et al. [35] interpret this effect as follows: participants with more severe hearing loss use strategies that are more efficient during a spatial working memory subtest. The authors suggest that this is caused by a more extensive use of working memory in daily life of hearing impaired to compensate the deficits that they have with auditory information. Following the authors, persons with hearing loss use working memory as compensatory mechanism. Similar results were reported by [34,36].

On the other hand, research shows that an impairment of speech comprehension [20] is accompanied by impaired short-term memory and memory in general. More recent theories consider impaired transfer from short-term memory to long-term memory as a partial function of the working memory due to losses in the acoustic perception [21,28,29,30]. Boxtel, M.P.J. [21] suggest “(…) that verbal memory function may be underestimated in individuals with mild to moderate degree of hearing loss.” (p. 152). The results of several studies suggest that information processing of people with reduced hearing capacity is often executed without error, but the information processing is associated with significantly increased effort. Often, the so called “effortfulness hypothesis” is used to explain these deficits in memory performance [37,38,39]. Following the “effortfulness hypothesis”, a hearing-impaired person needs more cognitive resources (higher performance effort) to achieve the same perceptual performance as a normal hearing person. According to this, more performance effort is needed by a person with reduced hearing capacity. This can result in reduced processing resources to encode the content and transfer it to short- and long-term memory. The effortfulness hypothesis is also supported by the results of research with normal hearing young adults, who had to perform a verbal memory task under different hearing conditions that simulate hearing impairment [40]. 

Often, studies with participants suffering from reduced hearing capacity investigate samples, which consist of elderly people or already retired former employees. Of course, it is known that the loss of hearing capacity increases markedly with increasing age, beginning in the fourth decade of life [41]. However, very little is known about older workers, as defined by the WHO [1]. Therefore, it is not clear whether the interplay between this age range and hearing impairment does have an influence on cognitive performance, and as a result, on job performance. Baltes and Lindenberger [42] showed a positive relationship between sensory functioning of hearing and vision, and intelligence for younger (25–69 years old) and older (70–103 years old) adults. However, the relationship seems to be stronger in old than in young age. Following [21], the association between hearing acuity and performance in a verbal memory task was not dependent on age (four age groups from 24 to 81 years). Gordon-Salant and Fitzgibbons [43] reported on an experiment on speech recognition that participants without hearing loss, no matter if they were younger (19–40 years old) or older (65–75 years old), performed better in all the tested hearing conditions than participants with hearing loss. Following the authors, the effect of hearing impairment was independent from age-effects in each of their analyses. Furthermore, following the results of [44], older participants with poorer hearing capacity (mild to moderate hearing loss) showed significant greater cost of dividing attention while recalling a world list (just-heard) when compared to younger and older participants with better hearing abilities (younger: 20–46 years old; older: 67–80 years old). This was the case, even though the groups did not differ regarding their basic cognitive abilities that were measured with cognitive measures for working memory, episodic memory, and executive control. To further extend past research, persons of working age were investigated in our study.

### 1.2. Long Working Hours

According to article 2 of the “Directive 2003/88/EC of the European Parliament and of the Council of 4 November 2003 concerning certain aspects of the organisation of working time” (p. L299/10) [45], “working time” is defined as “any period during which the worker is working, at the employer’s disposal and carrying out his activity or duties, in accordance with national laws and/or practice”. “Rest period” is defined as “any period which is not working time”. Generally, occupational scientists define “long working hours (LWH)” as working hours that are beyond normal weekly hours of work. According to this, no consistent number of weekly hours to define LWH can be found in literature (e.g., [46]: LWH = more than 40 working hours a week; [47]: LWH = more than 60 h a week). Under the terms of article 6 “Maximum weekly working time” of [45], it is stated that “the average working time for each seven-day period, including overtime, does not exceed 48 h.“ (p. L299/11). Therefore, this measure is also often used [48,49]. The European Directive does not define an exact limit for working hours of a working day, but a recovery time of eleven hours a day is prescribed. However, following [50], long working hours are prevalent within today’s working world. In addition, results of the “Foundation’s Third European Survey on Working Conditions” (15 EU Member States; [51]) showed that at least 17% of the full-time employees are affected by long working hours or rather worked more than 45 h a week. For this reason, long working hours are from our point of view a very important topic that has to be investigated further.

From a long-term perspective, long working hours or working overtime hours is associated with a broad range of negative effects, such as an average decrease in productivity and work performance, increased error rates, a negative impact on safety at work, and therefore an increased risk of accidents and on employees’ well-being, health and private life [46,52,53,54,55,56,57,58,59,60,61,62,63,64,65,66,67,68,69,70,71,72,73,74,75]. Furthermore, following different research groups, employees working long working hours show poorer performance in cognitive tasks, concentration, and attention [55,76]. However, as Kallus, Boucsein, and Spanner [77] pointed out, time is only one aspect of work schedules. The type of work schedule might be an important moderator, especially in shift work. This is confirmed by the results of Persson et al. [78] who compared a traditional 40-hours work schedule and 84-h work week with alternate weeks off, which was requested by the workers themselves. The results indicate no significant differences of performance, fatigue, and sleepiness, as well as normal HPA (hypothalamic–pituitary–adrenal) activation regarding the two variants.

Regarding age, the results of the “Foundation’s Third European Survey on Working Conditions” [51] indicate that older employees do not work shorter hours than younger employees. When considering demographic changes of the working world, studies with an age-related impairments-specific point of view investigating the effect of long working hours are of great importance.

Effects of hearing impairment and age on cognitive performance for example were investigated in different studies before. Through, the combined effects of long working hours, age (especially persons within working-age), and age-related impairments like reduced hearing capacity were not investigated in direct combination. If age was mentioned in the studies, the authors reported that age was controlled within their samples. Our study extends past research on long working hours by comparing different age groups instead of using age merely as a control variable. Until now, how to deal with possible age-related impairments of sensory functions, like hearing impairment and work performance during extended working time, has not been addressed explicitly. Moreover, hearing impairment is one of the prominent symptoms of increasing age. The current paper addresses this interplay in a laboratory study. Therefore, the aim of the study presented in this paper was to examine if age and age-related impairments, especially hearing impairment, do have an impact on work performance during long working hours. Accordingly, the following research question should be answered: Do age and (age-related) hearing impairment have an impact on the (work) performance of people working overtime hours?

## 2. Materials and Methods 

The laboratory study was conducted in an experimental laboratory at the Department of Psychology at the University of Graz, Austria. The experimental laboratory was designed like a normal office workplace (single office with computer workstation).

### 2.1. Participants 

In total 61 employees (white-collar workers) participated in the laboratory study. 55 out of 61 participants completed all of the required performance measures. Therefore, the final sample for analysis presented within this paper consists of 55 white-collar workers, aged between 24 and 63 years (*M* = 39.55, *SD* = 11.49). 52.73% of the participants were female and 47.27% were male. The WHO (1) defines “aging workers” or “older workers” as workers who are aged 45 years or older. The study participants can therefore be grouped as follows: 32 participants (58.18%) belong to the group of “younger workers” and 23 participants (41.82%) belong to the group “aging workers” or “older workers”. All of the participants performed their work primarily in office workplaces. 7.27% of them were self-employed, 20.00% worked in a leadership position. Most of the participants (56.36%) reported an average overtime of 1–5 h per week in the last three months before participating in the study, 23.64% reported an average overtime of 6–10 h per week, 10.91% of 11 and more hours per week. 9.09% of the participants reported that they did not work overtime hours in the last three months. None of the 55 participants of the final sample owned a hearing aid. Pure tone audiometry was used to screen participant’s hearing abilities. Participants’ hearing loss (HL) ranged from 4.17 to 47.50 pure tone average (PTA) dB HL (*M* = 12.94, *SD* = 7.34) for their poorer hearing ear. Participants were included in the hearing impaired group when the worse ear hearing loss was 15 dB (or more) on at least two out of the four speech relevant frequencies (0.5, 1, 2 and 4 kHz). According to this criterion, 24 participants (female: 15, male: 9; age: *M* = 44.79, *SD* = 12.19) were included in the hearing-impaired group and 31 participants (female: 14, male: 17; age: *M* = 35.48, *SD* = 9.19) were included in the normal hearing group. The unequal distribution of age of the two hearing groups, *t* (53) = −3.23. *p* = 0.002, might be expected based on the natural aging process of auditory functions. In contrast to this, no significant differences between the two groups can be found regarding their average overtime per week in the last three months for the number of overtime hours in the current working week in which they participated in the study, nor for the time between the end of their “normal” working day and the testing. This study was carried out in accordance with the recommendations of the ethics committee of the University of Graz with written informed consent from all of the participants (39/46/63 ex 2012/13). The participants were recruited via the homepage of the University of Graz, short articles in regional newspapers and notices that were posted at notice boards in different companies, supermarkets, universities, medical practices of otolaryngologists and hearing aid acousticians. All participants received 65 Euro to refund their transportation costs and as incentive for their participation.

### 2.2. Study Design and Materials

The study defined the following variables to answer the research question whether age and (age-related) hearing impairment do have an impact on work performance, especially in LWH using a 2 × 2 × 2 repeated measurement design. Independent variables: Age [younger employees (younger than 45 years), older employees (45 years and older)] and hearing impairment [normal hearing employees, hearing impaired employees (poorer ear hearing loss of 15 dB or more on a minimum of two out of the four (speech relevant) frequencies 0.5, 1, 2, and 4 kHz)]. The measurement repetition factor represents the time of measurement. Overall, the participants participated in the study for two consecutive days after “normal” working hours of the study participants (mean “normal” working hours before the participated in the study: day 1—*M* = 6.56, *SD* = 1.70; day 2—*M* = 6.52, *SD* = 1.77). Therefore, two times of measurement (*t1*: day 1; *t2*: day 2) were assessed for all of the dependent variables. The examination procedure is based on the “Grazer fatigue paradigm” [79]. The paradigm is used successfully for experimental studies of stress, and especially fatigue. On both days of the study, participants had to additionally “work” (performance tests etc.—Vienna Test System, SCHUHFRIED GmbH—simulated working situation) for at least three hours. This result in a simulation of long working hours (day 1: three to max. five additional hours; day 2: three additional hours). Following [80], a reliable measurement of fatigue may only be possible after a working duration of at least one to two hours. Therefore, a long period of execution was chosen for the presented study. Hence, the investigation was carried out after a “normal” working day of the participants. The time range of three to a maximum of five hours on day 1 can be explained due to the fact that the participants were divided into a “extended” and a “moderate” long working hour group in the original study and pooled for the analyses presented in this paper based on the absence of significant group-differences. Both of the groups performed the same tests and questionnaires in the same order, beginning with an introduction and a standard pure-tone audiometry, followed by a phase to provide socio-demographic data and execute different questionnaires (e.g., subjective emotional well-being, subjective evaluation of perceived fatigue etc., not part of this paper; see [81] for more details). Afterwards, a phase to familiarize with the Vienna Test System (SCHUHFRIED GmbH, Vienna, Austria) was conducted, followed by a working phases with performance tests using the Vienna Test System (see subsequent sections for more details). The only difference between the two groups was that the “extended” working group has to perform additional tests after the common part of the study. These additional tests are not part of the presented paper. The study procedure was repeated on day 2, with the modification that on day 2 all participants only worked “moderate” long working hours (three additional hours). The dependent variables (DVs) to answer the research question are: DV1: long-term selective attention and concentration, DV2: reaction time and DV3: attention and concentration. The three dependent variables were measured using the Vienna Test System (SCHUHFRIED GmbH), which includes several tasks and performance measures presented on screen. Subsequent, the different measures are discussed in more detail.

#### 2.2.1. Long-Term Selective Attention and Concentration

To measure attentional performance the DAUF-sustained attention S3: Normal form with seven triangles (3∇) in irregular jumps (short: DAUFS3; Copyright © 1992 by SCHUHFRIED GmbH) was used. The DAUFS3 scores two main variables: (1) Number of correct reactions (SUMR) and (2) Average reaction time of correct reactions (MTR); and two additional variables: (3) Number of false reactions (SUMF) and (4) Average reaction time of false reactions (MTF). In this study, all four of the variables (main and additional variables) were included in statistical analysis.

#### 2.2.2. Reaction Time

For measurement of reaction speed the Reaction-Test S5: Choice reaction, yellow/tone, yellow/red—reaction to critical stimulus combination (short: RTS5; Copyright © 1996 by SCHUHFRIED GmbH) was used. The RTS5 consists of several different tasks. Participants have to react as fast as possible to optical or acoustic signals. The RTS5 scores two main variables that are both included in statistical analysis in this study: (1) Mean reaction time (M-RZ) and (2) Mean motor time (M-MZ).

#### 2.2.3. Attention and Concentration

To measure attention and concentration the COGNITRONE S2: Parallel form 2 with free working time (short: COGS2; Copyright © 1995 by SCHUHFRIED GmbH) was used. The task within COGS2 is to compare geometric figures and decide whether the figure to be compared is identical to the geometric figure shown below on the screen. COGS2 Version scores the main variable mean time: correct rejection (MTRN).

#### 2.2.4. Pure-Tone Audiometry

A standard audiometer (Micromate 304, Madsen Electronics, Taastrup, Denmark) was used at the beginning of the first study session to conduct a pure-tone audiometry. Following the WHO [11] and the European Working Group on Genetics of Hearing Impairment (EUWG, [82]), hearing loss was measured by audiometry and calculated on the basis of the pure-tone average (PTA) of hearing thresholds at 0.5, 1, 2, and 4 kHz.

### 2.3. Statistical Analyses

The statistical analyses of data were conducted using the software SPSS for Windows (SPSS Inc., Chicago, IL, USA). The analyses were based on a significance level of 5%. 2 × 2 × 2 MANOVAs (multivariate analysis of variance) and a 2 × 2 × 2 ANOVA (analysis of variance) with repeated measures were chosen as statistical procedure. Error type 1 was adjusted for the three groups of dependent variables using the Bonferroni-Holm [83] procedure for each effect.

## 3. Results

The results for answering the research question: “Do age and (age-related) hearing impairment have an impact on (work) performance of people working overtime hours?” are presented below.

### 3.1. Long-Term Selective Attention and Concentration

A MANOVA with repeated measures revealed a significant effect for age, *F_HF_* (4, 48) = 4.61, *p* = 0.003, *η_p_*^2^ = 0.278, time of measurement *F_HF_* (4, 48) = 9.07, *p* < 0.0001, *η_p_*^2^ = 0.430 and the interaction time x hearing impairment, *F_HF_* (4, 48) = 2.83, *p* = 0.035, *η_p_*^2^ = 0.191. All other effects did not reach the level of significance. Following the univariate analyses, older participants reacted slower than younger participants, no matter if the answer was correct or false (average reaction time of correct reactions (MTR): *F* (1, 51) = 17.34, *p* < 0.0001, *η_p_*^2^ = 0.254, average reaction time of false reactions (MTF): *F* (1, 51) = 7.39, *p* = 0.009, *η_p_*^2^ = 0.127; mean values are presented in Table 1 and Table 2). Younger participants showed a greater number of correct reactions (SUMR), *F* (1, 51) = 5.02, *p* = 0.029, *η_p_*^2^ = 0.090, older participants showed a greater number of false reactions (SUMF), *F* (1, 51) = 12.82, *p* = 0.001, *η_p_*^2^ = 0.201. 

**Figure 1:**
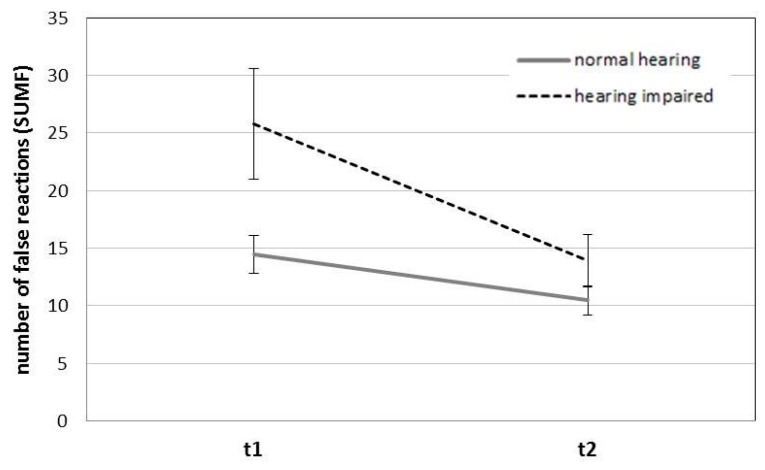
Long-term selective attention and concentration—number of false reactions: interaction hearing impairment x time of measurement. Note. Error bars reflect standard error of mean.

Furthermore univariate analysis revealed a significant interaction hearing impairment x time of measurement for the average reaction time of false reactions (MTF), *F* (1, 51) = 7.73, *p* = 0.008, *η_p_*^2^ = 0.132, and number of false reactions (SUMF), *F* (1, 51) = 5.77, *p* = 0.020, *η_p_*^2^ = 0.102. Hearing-impaired participants were slower when compared with participants in the normal hearing group at measurement time 1. This effect disappeared with the second time of measurement (see mean values in Table 2). Regarding the number of false reactions, hearing-impaired participants showed more false reactions in both times of measurement. The effect is most pronounced for measurement time 1 (see Figure 1).

### 3.2. Reaction Time

A MANOVA with repeated measures showed a significant effect for age, *F* (2, 50) = 4.03, *p* = 0.024, *η_p_*^2^ = 0.139, and a significant effect for time of measurement, *F* (2, 50) = 9.84, *p* < 0.0001, *η_p_*^2^ = 0.282. No other effects in reaction time did reach the 5%-level of significance. When considering both, the mean reaction time (M-RZ) and the mean motor time (M-MZ), older participants were slower than younger participants (M-RZ: *F* (1, 51) = 5.02, *p* = 0.029, *η_p_*^2^ = 0.090; M-MZ: *F* (1, 51) = 5.77, *p* = 0.020, *η_p_*^2^ = 0.102; for mean values see Table 3). Furthermore, participants reacted slower regarding both variables on the first day (*t1*) than on the second day (*t2*; M-RZ: *F* (1, 51) = 7.46, *p* = 0.009, *η_p_*^2^ = 0.128; M-MZ: *F* (1, 51) = 15.41, *p* < 0.0001, *η_p_*^2^ = 0.232).

### 3.3. Attention and Concentration

The results of an ANOVA with repeated measures showed a significant effect for time of measurement for the mean time correct rejection (MTRN), *F* (1, 51) = 6.81, *p* = 0.012, *η_p_*^2^ = 0.118. No other effects reached the 5%-level of significance. Results show that the participants needed more time for correct rejection at the first time of measurement (*t1*) when compared with the second time of measurement (*t2*). Mean values are shown in Table 4.

## 4. Discussion

Until now, how to deal with possible age-related impairments of sensory functions like hearing impairment and work performance especially, during extended working time, has not been addressed explicitly. Therefore, the aim of the study presented in this paper was to examine if age and hearing impairment do have a more than additive impact on work performance during extended working hours. Accordingly, the following research question should be answered: Do age and (age-related) hearing impairment have an impact on (work) performance of people working overtime hours? Following the “Grazer fatigue paradigm” [79] participants worked overtime hours after their normal working day. The three different performance tests that are presented in this paper represent a wide range to simulate and measure work performance. The three tests allowed us to measure general attention, long-term selective attention, concentration and reaction time. All of them represent important factors in the workplace.

Overall, the results show effects for age, the interaction of hearing impairment and time of measurement, as well as effects of the measurement time. First, regarding age the results for long-term selective attention and concentration show that older participants reacted slower than younger participants did, no matter if their answer was correct or false. In addition, following the results of the reaction test, the mean reaction time and the mean motor time of older participants were slower than the time younger participants needed. Furthermore, younger participants showed a greater number of correct reactions in the test for long-term selective attention and concentration, whereas older participants showed a greater number of false reactions. The results are in line with the results of other research groups who showed that fluid intelligence decreases with increasing age [84]. A further aspect in the field of cognitive abilities is the possibility of age-related changes in information processing, which are summarized under the term “general slowing” [85,86]. 

Second, an impact of hearing impairment can be shown as interaction with time of measurement for long-term selective attention and concentration. Hearing impairment seems to have a negative impact especially on measures of false reactions (frequency and reaction time). The results show, hearing-impaired participants were slower regarding their average reaction time of false reactions compared with participants in the normal hearing group at measurement time 1. This effect disappeared with the second time of measurement. Regarding the number of false reactions, hearing-impaired participants showed more false reactions in both of the times of measurement. The effect is most pronounced for measurement time 1. Both results can be interpreted as follows: Hearing-impaired participants are very fast in compensating their deficits. From our point of view, they have learned to react very fast to problems due to their reduced hearing capacity, which they may be faced frequently at their working place. In order to confirm this hypothesis, future studies shall include this aspect. Zekveld et al. [34,35] suggest as well that hearing-impaired persons use their working memory extensively to compensate their deficits in daily life. Further studies shall help to learn more about these compensatory mechanisms.

Third, regarding the effect of time of measurement the results for attention and cognition show that participants needed significantly more time for correct rejection at the first time of measurement compared with the second time of measurement. This effect does not match the expectations regarding the effect of the additional working hours that might result in fatigue, and therefore poorer performance on the second day of the study. Thus, training effects need special attention in research of aging workers and shall be part of future research.

### Study Limitations and Directions for Future Research

This study has some limitations. First, our study participants had to work long working hours on two consecutive days. From our point of view, it would be interesting to know whether the effects remain also valid for extended periods of long working hours. Further studies should therefore address this to receive more information.

Second, the study participants did perform the performance measures on two days with additional working hours. We therefore recommend that future research shall include days without extended working hours as control condition. Furthermore, it might be of interest for future studies to think about the inclusion of measures to include a general attitude regarding long working hours, as well as the motivation to work overtime hours.

## 5. Conclusions

In conclusion, the results of our study show effects for age, the interaction of hearing impairment and time of measurement, as well as effects of the measurement time. The results show that older participants reacted slower than younger participants did. Furthermore, younger participants reacted more frequently in a correct way. The results may be explained by a decrease of fluid intelligence in increased age and/or by a general cognitive slowing of older employees. Hearing impairment seems to have a negative impact especially on measures of false reactions (frequency and reaction time) and therefore especially on measurement time 1. The result that the effect is more pronounced for measurement time 1 than for measurement time 2 can be interpreted in a way that hearing-impaired participants are able to compensate their deficits over time.

## Figures and Tables

**Table 1:** Descriptive statistics—long-term selective attention and concentration.

Age	Hearing Impairment	MTR (s)				SUMR			
		*t1*		*t2*		*t1*		*t2*	
		*M*	*SD*	*M*	*SD*	*M*	*SD*	*M*	*SD*
Younger employees	Normal hearing	0.70	0.07	0.67	0.07	255.67	15.77	262.24	8.34
	Hearing impaired	0.71	0.06	0.68	0.06	255.89	19.54	261.00	13.96
	Total	0.70	0.07	0.68	0.07	255.73	16.64	261.87	10.10
Older employees	Normal hearing	0.78	0.05	0.75	0.06	250.75	12.45	252.50	9.77
	Hearing impaired	0.81	0.10	0.77	0.10	227.86	51.22	236.50	45.61
	Total	0.80	0.09	0.77	0.08	236.18	42.46	242.32	37.17
Total	normal hearing	0.72	0.08	0.70	0.08	254.31	14.88	259.55	9.65
	Hearing impaired	0.77	0.10	0.74	0.09	238.83	43.41	246.09	38.07
	Total	0.74	0.09	0.71	0.09	247.46	31.54	253.60	26.87

Note. *t1* and *t2*: Average reaction time of correct reactions (MTR) and number of correct reactions (SUMR).

**Table 2:** Descriptive statistics—long-term selective attention and concentration.

Age	Hearing Impairment	MTF (s)				SUMF			
		*t1*		*t2*		*t1*		*t2*	
		*M*	*SD*	*M*	*SD*	*M*	*SD*	*M*	*SD*
Younger employees	Normal hearing	0.70	0.08	0.70	0.11	13.81	9.75	8.86	5.67
	Hearing impaired	0.76	0.09	0.68	0.08	13.00	6.93	8.44	5.46
	Total	0.72	0.09	0.69	0.10	13.57	8.89	8.73	5.51
Older employees	Normal hearing	0.79	0.10	0.82	0.05	16.13	7.61	14.62	7.63
	Hearing impaired	0.82	0.11	0.76	0.16	34.00	26.77	17.36	12.33
	Total	0.81	0.10	0.78	0.13	27.50	23.25	16.36	10.74
Total	Normal hearing	0.73	0.10	0.73	0.11	14.45	9.14	10.45	6.66
	Hearing impaired	0.79	0.10	0.73	0.14	25.78	23.47	13.87	10.98
	Total	0.76	0.10	0.73	0.12	19.46	17.77	11.96	8.91

Note. *t1* and *t2*: Average reaction time of false reactions (MTF) and number of false reactions (SUMF).

**Table 3:** Descriptive statistics—reaction time *t1* and *t2*: mean reaction time (M-RZ) and mean motor time (M-MZ).

Age	Hearing Impairment	M-RZ (s)				M-MZ (s)			
		*t1*		*t2*		*t1*		*t2*	
		*M*	*SD*	*M*	*SD*	*M*	*SD*	*M*	*SD*
Younger employees	Normal hearing	544.68	70.16	506.18	67.20	140.59	35.03	132.14	48.74
	Hearing impaired	527.80	72.79	521.60	72.28	175.90	82.21	146.80	52.99
	Total	539.41	70.26	511.00	68.03	151.63	55.41	136.72	49.72
Older employees	Normal hearing	570.00	77.87	542.33	57.79	183.22	68.96	154.56	71.67
	Hearing impaired	593.07	84.63	575.36	99.11	217.57	73.24	195.50	64.46
	Total	584.04	81.06	562.43	85.38	204.13	72.06	179.48	68.85
Total	Normal hearing	552.03	72.11	516.68	65.80	152.97	50.14	138.65	56.03
	Hearing impaired	565.88	84.86	552.96	91.26	200.21	78.21	175.21	63.63
	Total	558.07	77.49	532.51	79.26	173.58	67.54	154.60	61.67

**Table 4:** Descriptive statistics—attention and concentration *t1* and *t2*: mean time correct rejection (MTRN).

Age	Hearing Impairment	MTRN (s)			
		*t1*		*t2*	
		*M*	*SD*	*M*	*SD*
Younger employees	Normal hearing	2.29	0.39	2.16	0.41
	Hearing impaired	2.28	0.37	2.17	0.32
	Total	2.29	0.38	2.16	0.38
Older employees	Normal hearing	2.43	0.25	2.38	0.30
	Hearing impaired	3.01	0.98	2.72	0.55
	Total	2.79	0.82	2.59	0.49
Total	Normal hearing	2.33	0.36	2.22	0.39
	Hearing impaired	2.71	0.86	2.49	0.54
	Total	2.49	0.65	2.34	0.47
